# Risk-taking propensity and emotional intelligence: an emotional version of the balloon analogue risk task (BART)

**DOI:** 10.1192/j.eurpsy.2024.1700

**Published:** 2024-08-27

**Authors:** A. Megías-Robles, M. Sánchez-López, R. Gómez-Leal, P. Fernández-Berrocal

**Affiliations:** Department of Basic Psychology, University of Málaga, Málaga, Spain

## Abstract

**Introduction:**

It is well known that emotions guide decision-making processes in risk contexts. Several studies in the literature have showed the influence of emotions on risk-taking using the Balloon Analogue Risk Task (BART).

**Objectives:**

The aim this research was to investigate the influence of emotional intelligence (EI) levels on the impact of emotions in risk-taking propensity assessed by the BART.

**Methods:**

To this end, we developed a variant of the BART in wich each balloon displayed a face with an emotional expression: happiness, fear, or neutral. EI was assessed from the performance-based ability model by the MSCEIT. The sample consisted of 120 participants (M_age_ = 21.52; 80% women).

**Results:**

A repeated measures ANOVA revealed a higher tendency to take risks when happy faces were presented, compared to the fear and neutral conditions. Moreover, participants with higher levels of EI showed a lower tendency to take risks across all emotional conditions. This relationship was particularly strong in the fear faces.

**Conclusions:**

Our findings support the effect of incidental emotions on risk-taking and suggest the role of EI as a protective factor for risk engagement.

**Disclosure of Interest:**

None Declared

